# Complex patterns of collective escape in starling flocks under predation

**DOI:** 10.1007/s00265-018-2609-0

**Published:** 2019-01-19

**Authors:** R. F. Storms, C. Carere, F. Zoratto, C. K. Hemelrijk

**Affiliations:** 1 Theoretical Research in Evolutionary Life Sciences (TRÊS), Groningen Institute for Evolutionary Life Sciences (GELIFES), University of Groningen, Nijenborgh 7, 9747AG Groningen, The Netherlands; 2 Department of Ecological and Biological Sciences, University of Tuscia, viale dell‘Università s.n.c., 01100 Viterbo, Italy; 3 Centre for Behavioural Sciences and Mental Health, Istituto Superiore di Sanità, viale Regina Elena 299, I-00161 Rome, Italy

**Keywords:** Collective motion, Flocking, Predation, Collective escape, Starling, Peregrine falcon

## Abstract

**Abstract:**

Collective behaviour of animals has been a main focus of recent research, yet few empirical studies deal with this issue in the context of predation, a major driver of social complexity in many animal species. When starling (*Sturnus vulgaris*) flocks are under attack by a raptor, such as a peregrine falcon (*Falco peregrinus*), they show a great diversity of patterns of collective escape. The corresponding structural complexity concerns rapid variation in density and shape of the flock over time. Here, we present a first step towards unravelling this complexity. We apply a time series analysis to video footage of 182 sequences of hunting by falcons on flocks of thousands of starlings close to two urban roosts during winter. We distinguish several types of collective escape by determining the position and movement of individuals relative to each other (which determines darkness and shape of the flock over time) as well as relative to the predator, namely ‘flash expansion’, ‘blackening’, ‘wave event’, ‘vacuole’, ‘cordon’ and ‘split’. We show that the specific type of collective escape depends on the collective pattern that precedes it and on the level of threat posed by the raptor. A wave event was most likely to occur when the predator attacked at medium speed. Flash expansion occurred more frequently when the predator approached the flock at faster rather than slower speed and attacked from above rather than from the side or below. Flash expansion was often followed by split, but in many cases, the flock showed resilience by remaining intact. During a hunting sequence, the frequencies of different patterns of collective escape increased when the frequency of attack by the raptor was higher. Despite their complexity, we show that patterns of collective escape depend on the predatory threat, which resembles findings in fish.

**Significance statement:**

Patterns of collective escape in flocks of starlings have always intrigued laymen and scientists. A detailed analysis of their complex dynamics has been lacking so far, and is the focus of our present study: we analysed video footage of hunting by falcons on flocks of thousands of starlings and show how patterns of collective escape (namely flash expansion, blackening, wave event, vacuole, cordon and split) depend on the preceding pattern and on details of attack. A higher frequency of attack during a hunting sequence resulted in a higher frequency of collective escape events. Flash expansion happened most often when the predator attacks at greater speed. A wave event was most likely when the raptor attacks at medium (rather than high or low) speed. These results provide a first quantitative approach to social complexity in collective avoidance of a predator.

**Electronic supplementary material:**

The online version of this article (10.1007/s00265-018-2609-0) contains supplementary material, which is available to authorized users.

## Introduction

Predator-prey relationships are the result of an evolutionary arms race in which the prey adopts strategies to reduce the risk of being captured (Dawkins and Krebs 1979). Grouping is one of the antipredatory strategies widely adopted across animal taxa (e.g. primates: Imanishi 1960; fish: Shaw 1978; birds: Feare 1984; cetaceans: Connor 2000; insects: Kastberger et al. 2008). Grouping has several benefits: it lowers the individual chance of being caught (dilution effect, Krause and Ruxton 2002) and decreases the area over which individuals risk being attacked (selfish herd hypothesis, Hamilton 1971). In groups, potential threats may be spotted earlier and information may be spread faster (collective detection and many eyes hypothesis, Krause and Ruxton 2002) than among solitary individuals. Grouping may also make it difficult for a predator to single out prey (confusion effect, Landeau and Terborgh 1986; Krause and Ruxton 2002; Kastberger et al. 2008; Hogan et al. 2017).

The collective motion of grouping organisms has been the subject of recent studies in multiple disciplines (e.g. Ioannou et al. 2012; Viczek and Zafeiris 2012; Jolles et al. 2017; Sumpter et al. 2018). In vertebrate taxa such as birds and fish, the highly coordinated motion and diverse collective displays have been studied both theoretically and empirically. In these studies, the patterns of collective motion have been explained as an emergent property from the interactions that occur at an individual level (Ballerini et al. 2008; Hemelrijk and Hildenbrandt 2011, 2012). It has been suggested that birds such as starlings interact with their six to seven closest neighbours, and that such interactions suffice to explain the general aspects of collective movement of flocks (Okubo 1986; Reynolds 1987; Heppner and Grenander 1990; Ballerini et al. 2008; Hildenbrandt et al. 2010). These interactions, combined with the flying behaviour of starlings, explain details of the internal structure of the flocks, measured as shape changes during turning (Pomeroy and Heppner 1992; Hemelrijk and Hildenbrandt 2011, 2012; Attanasi et al. 2013), stability of neighbours (Cavagna et al. 2013; Hemelrijk and Hildenbrandt 2015a) and degree of correlation in motion of neighbours at different distances (Bialek et al. 2014; Hemelrijk and Hildenbrandt 2015b; Cavagna et al. 2015).

The aforementioned studies focused primarily on undisturbed groups. However, the most complex patterns of collective motion occur under predation. Most of the research on collective escape induced by the predator has been conducted in fish (e.g. Nøttestad and Axelsen 1999; Gerlotto et al. 2006). One such a study concerned the relation between intensity of threat by a predator pike (*Esox lucius*) and patterns of collective escape of schools of minnows (*Phoxinus phoxinus*, Magurran and Pitcher 1987; Magurran 1990). Minnows compacted into a single school in the presence of the predator. When the pike was stalking, they showed skittering and group jumping behaviour (a series of rapid collective acceleration and deceleration). When attacked, the school showed flash expansions and fountains, followed by splits, after which individuals either merged back into the school or hid among stones.

While there are studies in taxa such as insects and birds that focus on specific patterns of collective escape (e.g. propagating waves, Kastberger et al. 2008; Procaccini et al. 2011, flash expansion, Romey and Lamb 2015), to our knowledge they did neither address how these patterns relate to each other nor to the acts of the predator. That is what we investigate in the present study for the first time for patterns of collective escape displayed by flocks of European starling (*Sturnus vulgaris*) in response to a raptor (Tinbergen 1951; Feare 1984; Carere et al. 2009). These patterns are widespread (Goodenough et al. 2017), but their function and underlying mechanisms remain debated (Carere et al. 2009; Zoratto et al. 2010; Procaccini et al. 2011). Our study focuses on the following questions: (i) which patterns of collective escape are displayed? (ii) how do these patterns inter-relate? and (iii) how do they depend on the behaviour of the predator?

## Material and methods

### Study species, areas and observations

During two winter seasons (January–March 2006 and December–March 2006–2007), data were collected on flocks of European starlings at two urban roosting sites (Termini and Eur) in Rome, Italy. These roosting locations are spaced about 10 km from each other and are both regularly visited by peregrine falcons (*Falco peregrinus*). Termini is situated in the city centre, and the roost there was used by up to 20,000 starlings. This site was under a relatively low predatory pressure and experienced regular hunts of only two peregrine falcons. The other roosting site (Eur) is located in the southern part of Rome, and was used by about 60,000 starlings and five peregrine falcons during the study period (Carere et al. 2009; Zoratto et al. 2010; Procaccini et al. 2011).

Video recordings were conducted at both roosting sites. Fifty-three recording sessions were executed between 14 January and 17 March in 2006 and 57 sessions between 12 December 2006 and 2 March 2007. Recording started 90 min before dusk and lasted until nightfall. A high-definition video camera (JY-HD10, JVC, 30fps) and MiniDV digital tapes were used. Two operators (CC and FZ) stood at a fixed location in front of the roost. In total, these recordings comprise 16 h of footage. Aerial manoeuvres of starling flocks, occurring as collective responses to the falcon, were recorded whenever possible.

### Video analysis and data extraction

All video materials were digitalized in MP4. Sequences in which the falcons were hunting a flock were selected. We defined a hunting sequence from when the falcon starts to pursue the flock until it either catches a starling or withdraws from the flock (Procaccini et al. 2011). From 182 recorded sequences, we measured the predation success of the falcon, the number of predators involved and the flock size and classified the escape responses. We then selected the hunting sequences in which the flock and falcon were clearly visible throughout for detailed analysis on the type of attacks and collective escape responses. Consistent independent observations by two observers (CC and RFS) led to the selection of 67 sequences for analysis.

**Fig. 1:**
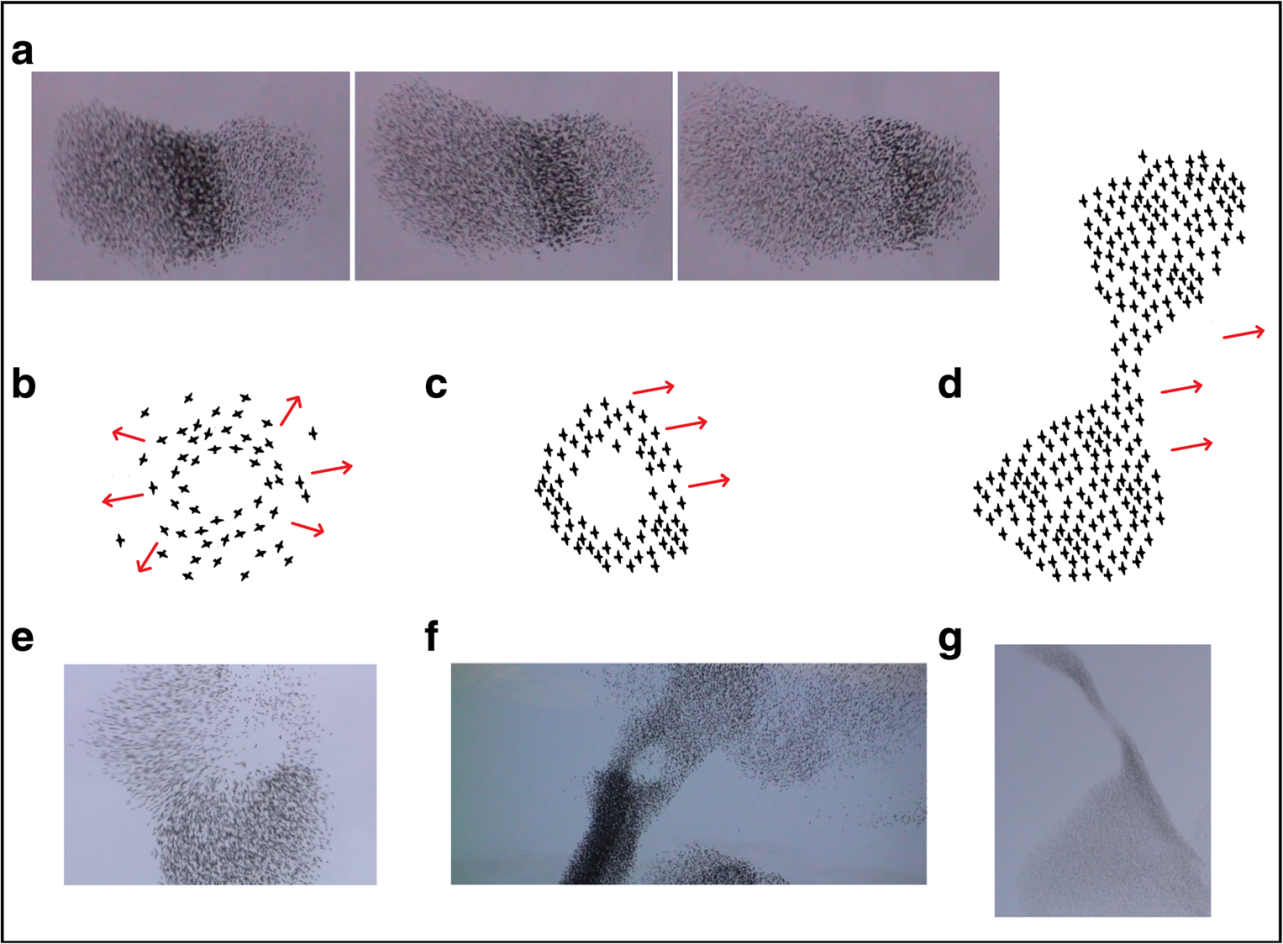
Video still images of collective escape patterns (**a**, **e**, **f**, **g**) and caricature of supposedly underlying behavioural acts (**b**–**d**). **a** Wave, showing one pulse progressing to the right through the flock. **b**, **e** Flash expansion. **c**, **f** Vacuole. **d**, **g** Cordon

To minimize observer bias, blinded methods were used to score all behavioural data. Each hunting sequence was examined on a frame-by-frame basis, noting the behaviour of the flock and falcon (see Table 1 and Fig. 1). The classification of behaviour during a hunting sequence was based on frequently observed patterns that were clearly identifiable, and although it covers a broad spectrum of the collective behaviour seen during a hunting sequence, it is not necessarily complete: more patterns of collective motion may be classified and some of the classified patterns may share a common underlying mechanism (Inada and Kawachi 2002).

**Table 1:** Description of each type of behaviour of the flock and the falcon

Behaviour/collective event	Description
Behaviour of falcon	
Attack	A manoeuvre approaching the flock aimed at catching prey
Collective pattern of flock	
Wave event	One or more pulses of optically darkened bands propagating through the flock (Procaccini et al. 2011) (Fig. 1a)
Blackening	The flock, or a part of it, darkens
Flash expansion	Starlings suddenly move radially outward from the flock (Fig. 1b, e)
Vacuole	For a certain period, in the flock in which flock members are aligned, there is a hole (Fig. 1c, f)
Split	Single flock splits into multiple subflocks
Merge	Multiple subflocks merge together
Cordon	Two relatively large parts of the flock are interconnected by a thin string of individuals (Fig. 1d, g)
Flock dilution	A flock spreads out and becomes lighter in colour, indicating larger distances between individuals.

For attacks, we determined their onset with high precision as a clear switch of the falcon’s on-going trajectory to one aimed at catching a prey. We classified attacks in terms of speed (slow/medium/high) and location relative to the flock (below/side/above). For this classification, we only included attacks that occurred primarily in the two-dimensional plane (vertically or horizontally). We distinguished between attacks that followed each other fast (referred to as repetitive attacks) from attacks that were isolated in time (referred to as isolated attacks) based on an inter-attack time of 5 s. The threshold of 5 s came from the distribution of inter-attack times (Fig. S1). We measured the duration and frequency of wave events, blackening and cordons and the frequency of split and merge events (Fig. 1, Table 1). Independent measurements of the number of pulses within a wave event as well as the relative location and speed of attacks taken by two observers (CC and RFS) agreed with one another. Some behaviours of the flock and falcon overlap in time (Fig. S2 and Online Resource 1).

For wave events that showed two or more pulses, we analysed the duration of the interval between pulses. The frames showing the wave event were analysed using ImageJ (Abràmoff et al. 2004; Schneider et al. 2012, Supplementary material). We also measured the interval between pulses via visual observation (see Supplementary material).

### Data analysis and statistics

#### Falcon-flock interactions: behavioural correlations and transitions

We investigated the order with which different collective events and attacks of the predator followed each other within an interval of 5 s; the duration of this interval is a conservative estimate of the time within which collective events and attacks are related to one another. A one-tailed permutation test was used to determine which transitions occur more often than expected by chance. Using Patefield’s algorithm, 100,000 transition tables were generated, showing the predicted occurrence of transitions by chance given the total number of transitions observed among each combination of different events (Patefield 1981).

The time it took for flock dilution to occur after an attack was 15.0 ± 2.4 s (mean ± SEM). Using a chi-squared test, we tested whether certain collective escape responses occurred more often than expected by a random distribution of events before or after an attack within a hunting sequence. The null hypothesis was that there is no relationship between the occurrence of attacks and collective escape patterns within a hunting sequence; thus, the distribution of collective escape patterns through time is unaffected by the timing of the attacks, which would make them random. For all statistics, we applied a Bonferroni correction. General linear models were used to examine correlations among behaviours of the flock and the falcon. In one of these models, the log-transformed frequency at which collective escape responses occurred within a hunting sequence was the response variable and the candidate explanatory variables were attack frequency, collective escape type, roosting site and the success of the falcon, using a Gaussian error distribution. Other models were used to test the effects of attack strategy, including explanatory variables such as attack speed and location, and roosting site on the probability of each of the specific collective responses occurring within or after 5 s of an attack. For these models, we used a logistic regression. Selection of the best model was based on the Akaike information criterion (AIC) scores, where models with a Δ_*i*_(AIC) higher than 2 were considered as significantly worse than the best model.

#### Analysis of the wave and predation success

The intervals between subsequent pulses of a wave event were measured with ImageJ (see Supplementary material) by measuring changes in the luminance in the same spot of a flock. We compared this result to the one obtained by directly observing the number of pulses and dividing by the duration of the wave event (see Supplementary material). Both methods were compared by *t* test for independent samples (see Supplementary material). The number of patterns of collective escape occurring in a sequence per attack was tested for differences between successful and unsuccessful hunting sequences using a general linear model. All analyses were performed with R (R Core Team 2016).

##### Data availability

The datasets generated during and/or analysed during the current study are available from the corresponding author upon request.

## Results

### Classification of patterns of collective escape

**Fig. 2:**
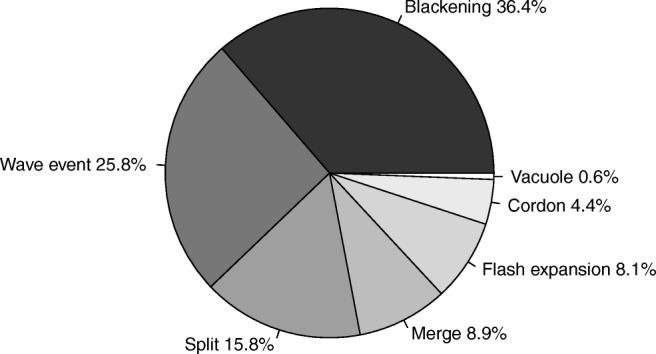
Pie chart of the percentage of different patterns of collective escape

A total of 795 collective events of flocks and 210 attacks by the falcon have been recorded. We identified seven types of collective escape (Fig. 2). Blackening was the most common (*N* = 289) and vacuole the least common (*N* = 5) (see Table S1).

### Falcon-flock interactions: behavioural correlations and transitions

**Fig. 3:**
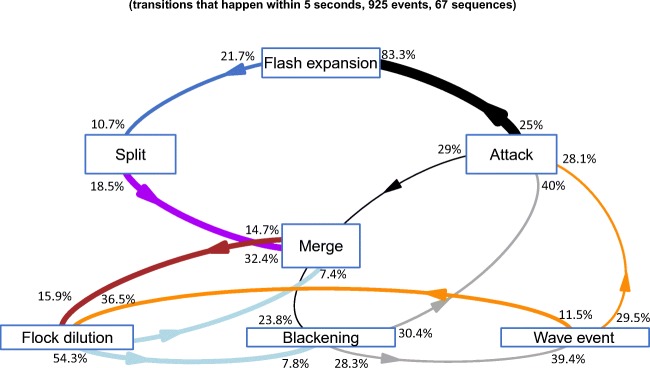
Summary of the order of different types of collective patterns of the flock and attack by the falcon. This figure shows the transitions between events within a timescale of 5 s. Arrows are only shown for transitions between patterns that significantly differed from what would be expected if they occurred randomly. The thickness of the arrows shows how often a transition occurs compared to what would be expected randomly. The numbers next to the arrows show the percentage of the starting event and the percentage of the following event a transition covers. For instance, 25% of all attacks were followed by a flash expansion and 83.3% of the flash expansions were directly preceded by an attack

Transitions between patterns of collective behaviour are shown in Fig. 3 with the corresponding *p* values in Table 2. Flash expansion occurred in response to an attack and was followed by splits. Merges happened after splits, followed by flock dilution. Blackening occurred before as well as after an attack, and also preceded wave events. Wave events preceded attacks and a flock dilution. Flock dilution was a precursor to merges and blackening.

**Table 2:** Statistical outcome of the permutation analysis of the temporal events of the flock and behaviour of the falcon. See “Material and methods” and Fig. 3 for further details

		*P* value						
Subsequent collective event and attack of the predator	Attack *n* = 200	0.99999	*< 0.00001*	*0.00044*	0.96001	0.98787	0.80523	0.98317
	Blackening *n* = 276	*0.04124*	1	0.38344	0.39109	*0.00001*	0.12986	0.21886
	Wave event *n* = 200	0.9252	*0.00002*	1	0.06604	0.56257	0.73161	0.07809
	Flash expansion *n* = 60	*< 0.00001*	0.99997	0.99983	0.97898	0.88975	0.92708	0.94399
	Flock dilution *n* = 35	0.99796	0.60222	*0.00044*	0.74658	0.90116	*0.00229*	0.64408
	Merge *n* = 68	0.99986	0.73906	0.06791	0.92892	*0.0271*	0.69967	*< 0.00001*
	Split *n* = 119	0.66058	0.85998	0.15307	*0.00875*	0.63384	0.30349	0.96887
		Attack *n* = 200	Blackening *n* = 276	Wave event *n* = 200	Flash expansion *n* = 60	Flock dilution *n* = 35	Merge *n* = 68	Split *n* = 119
		Preceding collective event and attack of the predator						

The entries shown in italics are the ones that occur significantly more often than predicted by chance

**Fig. 4:**
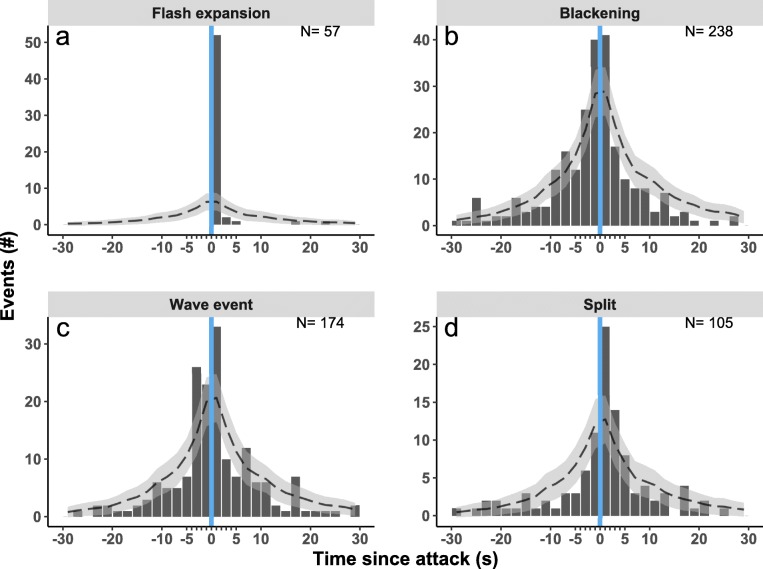
Frequency distribution of the shortest intervals between an attack and four patterns of collective escape. The *x*-axis shows the time before an attack (−) and after an attack (+) in seconds, and the *y*-axis shows the frequency. The dotted line indicates the expected occurrence, would events occur randomly in time, the grey ribbon shows the standard deviation

As to the relation in time between attacks by the raptor and patterns of collective escape, flash expansion was the only event occurring solely after an attack and never before it, *χ*^2^(29, *N* = 30) = 365.14, *p* < 0.001 (Fig. 4). Blackening occurred more often than expected between 4 s before and 2 s after an attack, *χ*^2^(29, *N* = 30) = 52.849, *p* = 0.004 (Fig. 4). Wave events and splits did not differ significantly from chance in their frequency of occurring before and after attacks.

**Fig. 5:**
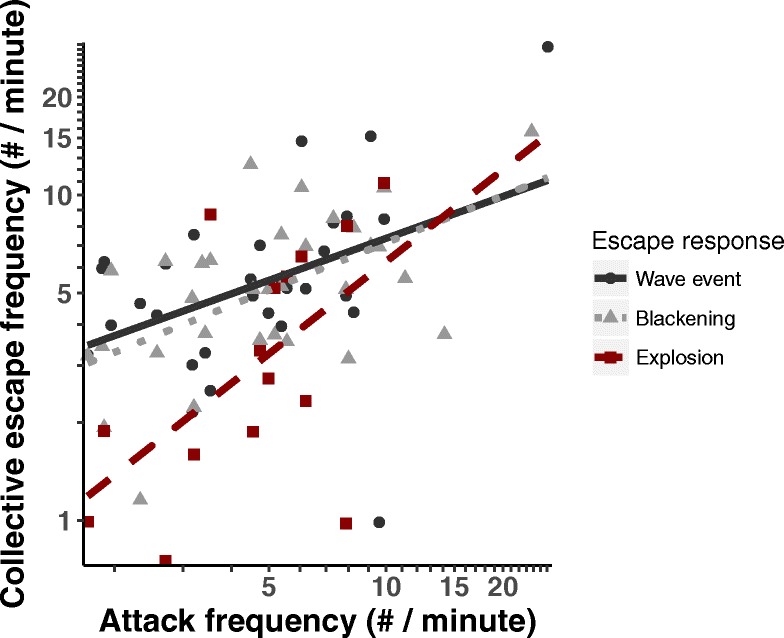
Relationship per hunting sequence between the frequency of attack and frequency of each of three collective escape patterns

Hunting sequences with a higher frequency of attack were associated with a higher frequency of blackening, wave events and flash expansion (Fig. 5, Table 3). The falcon mostly attacked from above the flock (*N* = 121), sometimes from the side (*N* = 36) and seldom from below (*N* = 18). It mostly attacked at medium speed (*N* = 144), in 16 cases at low speed and in 15 cases at high speed. Fifty-five of 175 attacks occurred within 5 s before or after another attack.

**Table 3:** General linear model on the log frequency of patterns of collective escape occurring during a hunting sequence. The AIC scores are shown, as well as the AIC differences Δ_*i*_(AIC), the likelihood of a model *L*_*i*_, the Akaike weight *W*_*i*_(AIC), the accumulative Akaike weight acc *W*_*i*_(AIC) and the evidence ratio (ER). The explanatory factors tested included attack frequency, escape response type, whether the raptor was successful in catching a prey and roosting site

Analysis of the frequency of collective escape per hunting sequence							
Model	df	AIC_*i*_	Δ_*i*_(AIC)	*L*_*i*_	*W*_*i*_(AIC)	acc *W*_*i*_(AIC)	ER
Attack frequency + pattern of collective escape	5	137.24	0	1	0.66	0.66	1
Attack frequency × pattern of collective escape	7	138.73	1.5	0.47	0.31	0.97	2.11
Attack frequency	3	143.74	6.51	0.04	0.03	1	25.88
1 (Null model)	4	156.33	19.1	0	0	1	14,018.42
Roosting site	2	161.23	23.99	0	0	1	162,038.51
Capture success	5	162.91	25.67	0	0	1	374,857.21

**Fig. 6:**
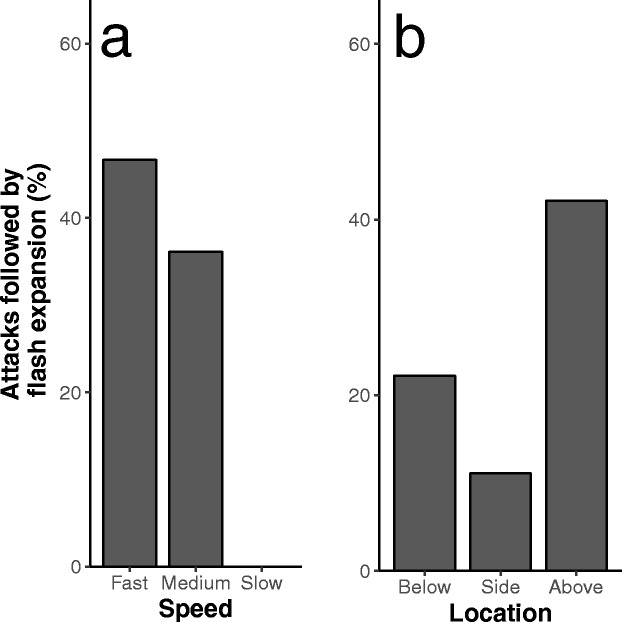
Probability of flash expansion per attack strategy. These figures show the probability of a flash expansion occurring within 5 s after an attack for attacks at different speeds and locations relative to the flock

The probability of a flash expansion occurring immediately after an attack (within 5 s) was highest when attacks were done at high speed rather than medium or low speed and when they came from above rather than from the side or below (Fig. 6, Table S2). Wave events were more common before and after attacks of medium speed rather than attacks of low or high speed (Table S3). The attack type did not impact the probability of blackening occurring before or after an attack. Splits were more probably to occur before and after attacks in roost Eur.

### Wave events

**Fig. 7:**
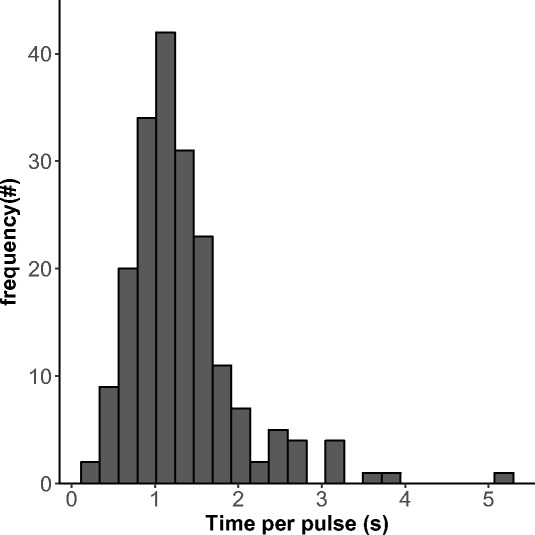
Frequency distribution of interpulse time (time it took for a pulse to be followed up by another pulse)

On average, wave events occurred 12.6 s after an attack and 13.9 s before an attack (*n* = 54). Wave events lasted 3.5 ± 0.23 s (mean ± SEM) and comprised 2.88 ± 0.19 pulses (mean ± SEM, 133 pulses in total). The time between pulses was on average 0.86 ± 0.44 s when estimated with ImageJ (Fig. 7).

## Discussion

Our empirical study helps understanding the complex time order of different patterns of collective escape and predator attack (Kappeler et al. 2019). We distinguished six patterns of collective escape by starling flocks in reaction to the peregrine falcon, namely blackening, wave event, split, flash expansion, cordon and vacuole in order of decreasing frequency. The frequency and type of pattern of collective escape was shown to depend on the intensity of threat by the predator. When the predator was absent or not in the vicinity, so presumably in situations of low threat, starling flocks were spread out with relatively large distances between individuals. When the predator was in the vicinity or actively pursuing the flock, presumably in situations of medium threat, flocks collectively displayed blackening and wave events. When the falcon attacked, clearly the case of high threat, flocks exhibited flash expansions followed by splits. Besides, the higher the frequency of attacks during a hunting sequence, the higher the frequency of blackening, wave events and flash expansion. A similar relation between patterns of collective escape and the degree of threat by the predator was also found in fish (Magurran and Pitcher 1987; Magurran 1990).

As to the relation in time between a collective escape and an attack, only flash expansion happened immediately after an attack, following an attack four to ten times faster than any of the other patterns of collective escape. Blackening and wave events occurred not only after an attack but also before it (Fig. 6). As regards the act of the predator triggering certain types of collective escape, flash expansion happened more often when the predator approached the flock at a relatively high speed and attacked from above rather than from the side or below. Wave events were more likely to occur before and after attacks of medium speed compared to attacks of low and high speed, and blackening events did not depend on the type of attack (in terms of its speed, location, repetitiveness, see Table S4). Splits were more likely to occur before and after attacks in only one of the two roosts (Eur), which could be due to the higher hunting pressure and larger flocks at this location (Table S5, Carere et al. 2009).

The effectiveness of collective escape to prevent being caught was hard to estimate: whereas in our earlier work, the wave event was shown to reduce the falcon’s capture success (Procaccini et al. 2011), in this study attacks with and without hunting success were equally likely to be preceded by a collective escape. The difference may be explained by our smaller sample of video footage and the lack of inclusion of direct observations in the field, which contributed to a higher sample size in the previous study.

A possible risk of flash expansions is that they reduce group cohesion and lead to splits (Fig. 3). The subsequent subflocks are smaller than the original flock, which facilitates targeting of individual prey by the predator. This may explain why flocks appeared resilient against splitting after 78.3% of the flash expansions and did not split (see Online Resource 2). A similar resilience has been reported for fish (Pitcher and Wyche 1983). Note that repetitive attacks may prevent individuals from returning to a flock after a flash expansion (see Online Resource 2). The trade-off between escaping the predator and returning to the group may be at the core of some of the observed escape patterns, and these may depend on the degree of threat of the predator.

In most of our footages (particularly those with blackening and waves), an individual-level analysis was not possible. Thus, the mechanisms underlying these escape responses cannot be determined conclusively in starling flocks with our data. In fish schools, blackening was shown to result from compacting (Magurran and Pitcher 1987; Magurran 1990). Combining models and empirical data, wave events are likely caused by individuals making zig manoeuvres of rolling sideward and back that increase their wing area visible to observers (Procaccini et al. 2011; Hemelrijk et al. 2015). Modelling studies of fish schools have shown that simple avoidance behaviour of fleeing ahead of the predator can lead to a variety of escape responses such as flash expansions, vacuoles, fountain events and herding (Inada and Kawachi 2002).

We acknowledge that our analysis has a number of limitations. The orientation of the flock may affect how we perceive patterns of collective escape. Blackening and wave events may be confused depending on the position of the observer, potentially leading to an overestimation of the number of events of blackening and underestimation of wave events. However, the other patterns of collective escape (splits, merges, flash expansions, cordons, vacuoles and flock dilution) are clearly distinct from one another and could not be confused. Our observations were conservative, in the sense that we may have missed some of the collective patterns if they were hidden to the observer by overlapping flocks in between the observer and the collective event. Note that our observations were continuous, and we selected only footage where the flock and the falcon were clearly visible (omitting unclear footage).

Another potential limitation of our study is that falcons experience patterns of collective escape differently from an observer because of their closer spatial location relative to the flock. Indeed, future work focusing on the viewpoint of the raptor (e.g. with the use of drones) is of great importance to gain information on how collective escape patterns are perceived by the falcon.

In sum, we have shown that the pattern of collective escape in starling flocks depends on the specifics of the hunting behaviour of the falcon and on the preceding pattern of collective escape. Such complex and dynamic patterns of collective escape emerge from the interplay between the antipredatory actions of thousands of prey and the hunting behaviour of the predator. These dynamics show similarity with collective patterns of escape in fish, suggesting that despite being complex, general rules may underlie these systems.

## Electronic supplementary material

ESM 1 (MP4 42,437 kb)

ESM 2 (MP4 20,589 kb)

ESM 3 (DOCX 1203 kb)
