# Supplementary material for: Complex patterns of collective escape in starling flocks under predation
Source: Behav Ecol Sociobiol. 2019 Jan 19;73(1):10. doi: 10.1007/s00265-018-2609-0 (PMC6404399; doi:10.1007/s00265-018-2609-0)
Supplement: Supplementary file 3 — (DOCX 1203 kb) [file 265_2018_2609_MOESM3_ESM.docx]

Supplementary material

## Complex collective motion: escape patterns in starling flocks under predation

Behavioral Ecology and Sociobiology

R.F. Storms^1*^, C. Carere^2^, F. Zoratto^3^, C.K. Hemelrijk^1^

^1^University of Groningen, Groningen Institute for Evolutionary Life Sciences (GELIFES), The Netherlands

^2^ University of Tuscia, Italy

^3^National Institute of Health, Italy

^*^Corresponding author: [r.f.storms@rug.nl](mailto:r.f.storms@rug.nl)

Theoretical Research in Evolutionary Life Sciences (TRES), GELIFES, University of Groningen,

Nijenborgh 7,

9747AG Groningen,

The Netherlands

Tel 0031-50-3636990
Fax 0031-50-3633400

**Table of contents**

1 Inter attack times

2 Actogram of a hunting sequence

3 Analysis of wave events

3.1 ImageJ analysis

3.2 Visual analysis

4 Variability of patterns of collective escape

5 Generalised Linear Models on the probability of escape responses

- 1. Flash expansion

5.2 Wave events

5.3 Blackening

5.4 Splitting

6 Online resources

6.1 Online resource 1

6.2 Online resource 2

**1 histogram of inter attack times**


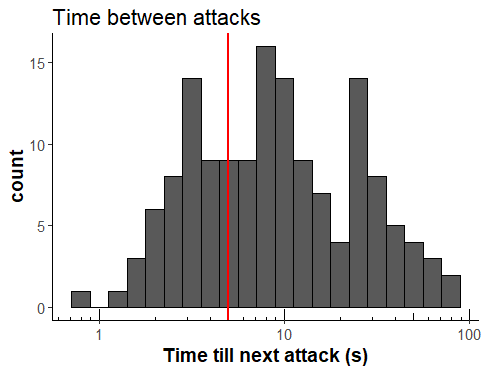


**Fig. s1** Histogram of the time inbetween attacks. The red line indicates where attacks occur five seconds after another. All attacks with an inter attack time below five seconds have been defined as repetitive attacks and attacks with an inter attack time longer than five seconds have been defined as isolated.

**2 Actogram of a hunting sequence**


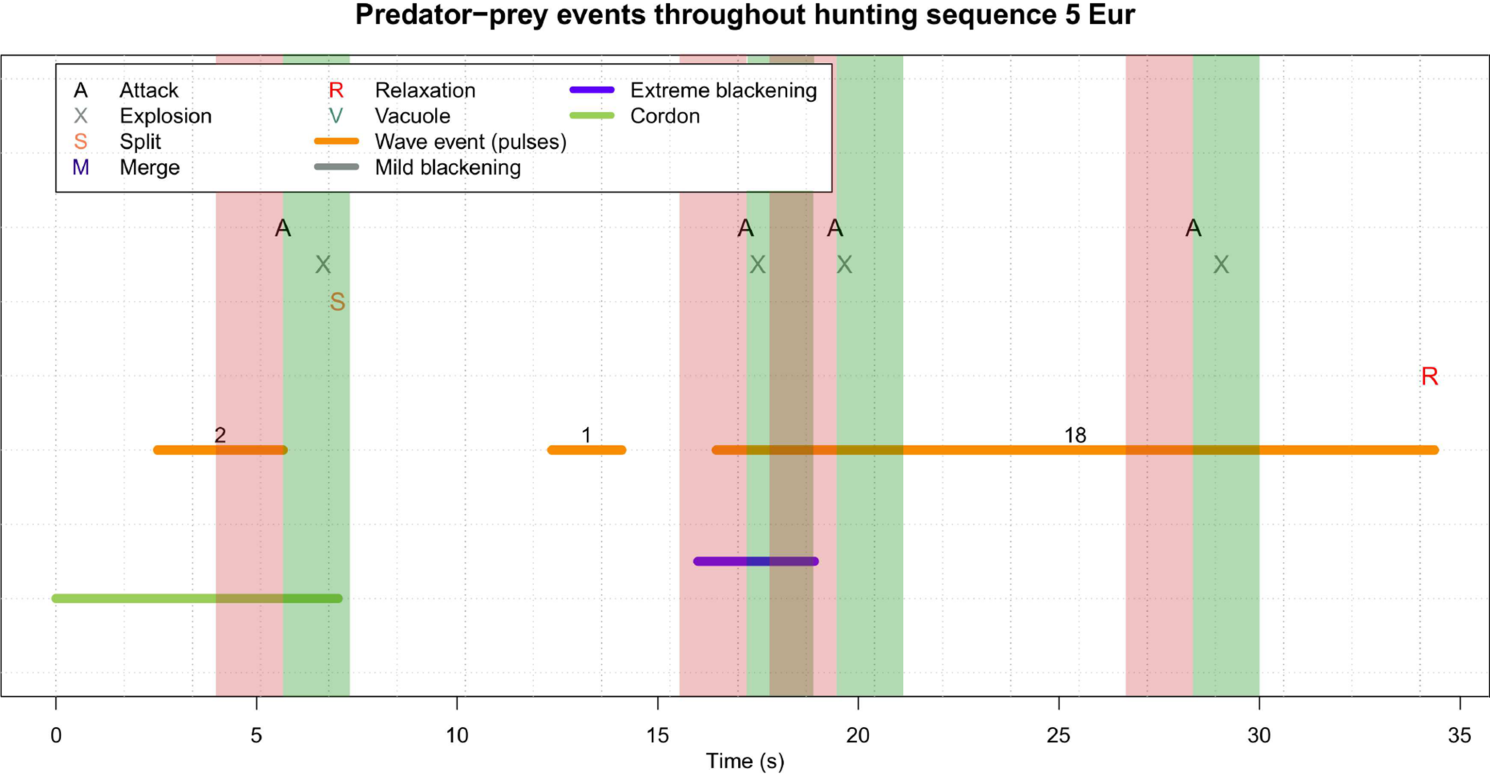


**Fig. s2** Actogram of a hunting sequence. This actogram shows the behaviour of the flock and falcon during a hunting sequence at roosting site Eur (Rome, Italy). The footage of the hunting sequence can be found in Online Resource 1, of which the seconds match to this actogram. The green overlay indicates a post-attack interval and the red overlay indicates pre-attack intervals in which flock events have been measured. So attacks occur at 6, 17, 19 and 28 seconds.

### 3 Analysis of wave events

**3.1 ImageJ analysis**


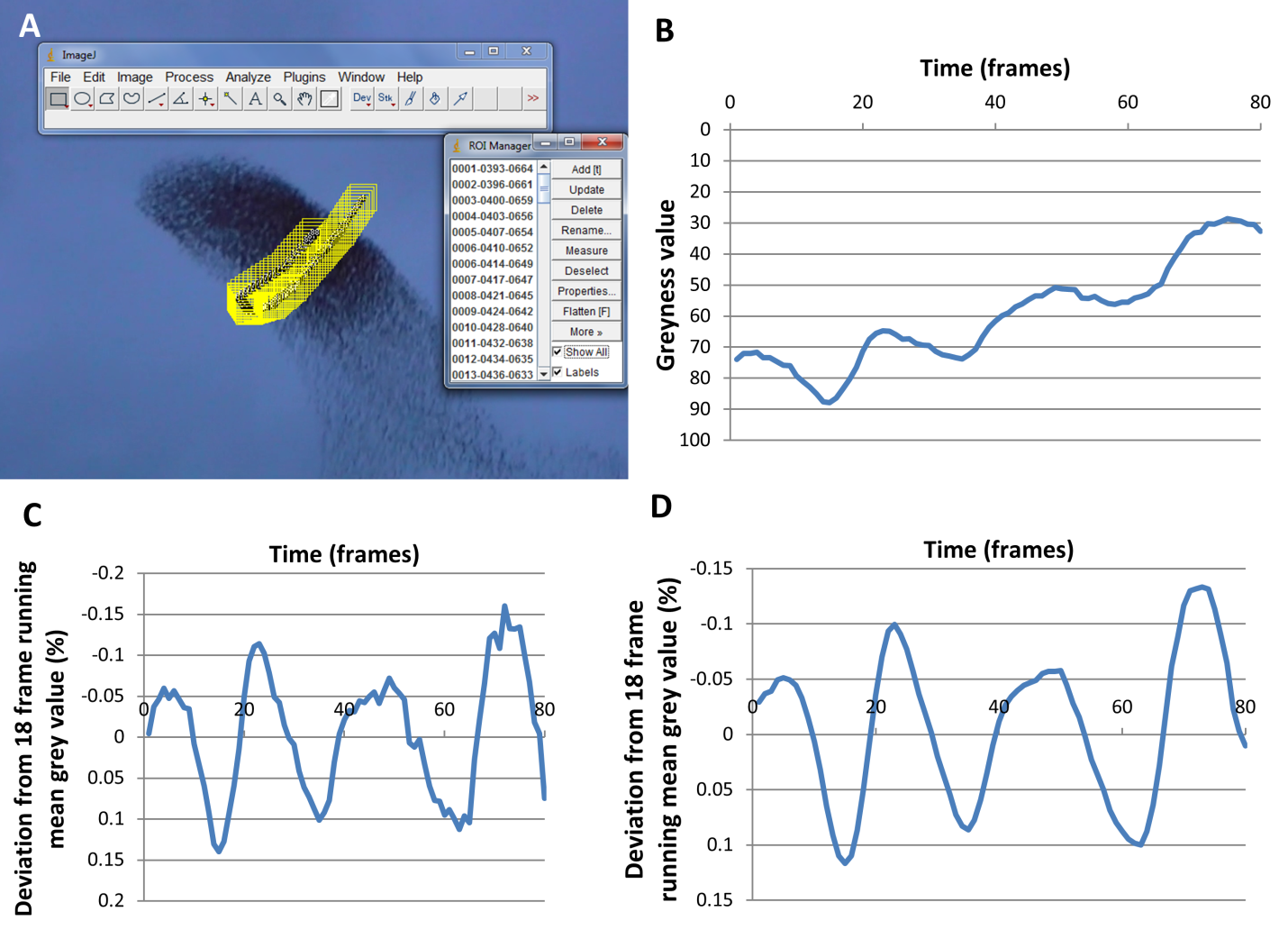


**Fig. s3** Example of analysis of wave events. **A**: ImageJ shows a region of interest (ROI) in yellow being followed for 80 frames. The background shows one of the frames from this wave event. For each frame, the luminance is calculated in the corresponding region of interest. Note that a second lasts 30 frames. **B**: The uncorrected luminance of the region of interest over time. **C**: The percent devation in luminance measured corrected for a 18 frame sized running mean. **D**: The smoothened percentage deviation in luminance.

The footage of sequences that concerned wave events with more than 2 pulses have been used for analysis of the pulse frequency and rhythmicity per event. The main focus was exploring the time it takes for a pulse to be followed up by another pulse.Using KMPlayer, the frames including the wave event were extracted from the footage. These frames were subsequently uploaded for each wave event as a stack in ImageJ (Abràmoff et al. 2004; Schneider et al. 2012). A region of interest and timeframe was chosen through which the pulses of the wave event were clearly visualized. The subset of the flock in this region was followed through time by manually identifying its location every 10 frames. The location of the region of interest in the intermittent frames was interpolated from the manually defined locations (Figure s2A). Over the course of the wave event the luminance was measured in the region of interest; if a pulse passes through the region of interest this is reflected in a decrease in luminance (Figure s2B). As the wave event sequences contain noise in luminance due to background changes and flock compacting and relaxation, this needs to be filtered out. It is important to note that pulses during a wave event occur in a far shorter timescale than the luminance changes happening due to noise. To correct for noise we subtract a running average of luminance at a large timescale x, leaving only the effects of the shorter timescale. The luminance was standardized between wave events by dividing the value of each frame by the running average as well, leaving the percentage deviation from the average luminance (Figure s2C):

$Lcorr(t)=\left(\frac{L(t)}{\frac{1}{x}\sum_{t-\frac{1}{2}x}^{t+\frac{1}{2}x} L}-1\right)*100\%$ (1)

The large timescale x has been set at 18 frames, which proved to be an accurate method of removing long term background noise while retaining the short term luminance effects of pulses moving through the area of interest. To correct for small stochastic changes, a smoothening was used over a frame timescale y:

$LSmooth(t)=\frac{1}{y}*\sum_{t-\frac{1}{2}y}^{t+\frac{1}{2}y} Lcorr$ (2)

The smoothening timescale y has been set to 5 frames. The resulting luminance, now shown in percentage deviation, solely shows the effects of the pulse passing through the area of interest (Figure s2D). The onset of a pulse was classified as an intercept with the x-axis with a decreasing luminance. Using these intercepts the time between the start of a pulse and the start of the next pulse was calculated and the maximum intensity of each pulse.

**3.2 Visual analysis**

This method focused on measuring the amount of pulses during a wave event by eye and dividing this by the duration of the wave event. The time between pulses was on average 1.33 $\pm$ 0.67 s, longer than what was measured with ImageJ. This difference can be explained by the duration of a wave event being measured using both the beginning location and the end location of a pulse rather than focusing on a single location.


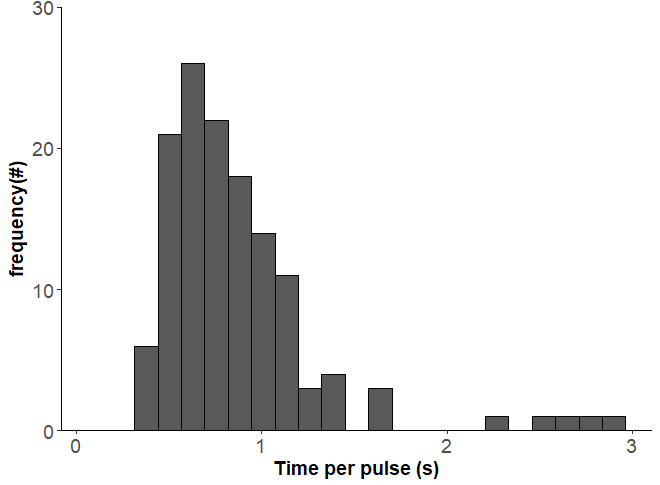


**Fig. s4** Frequency distribution of interpulse time (time it took for a pulse to be followed up by another pulse) using an observational analysis.

**4 Variability of patterns of collective escape**

| Event | Frequency (#) | Duration (s) | Time preceding attack (s) | Time after attack (s) | Frequency successful predation (#) | Frequency unsuccessful predation (#) |
| --- | --- | --- | --- | --- | --- | --- |
| Attack | 210 | - | - | - | 63 | 98 |
| Wave event | 205 | 3.48 ± 3.25 | 13.91 ± 15.99 | 12.62 ± 16.99 | 53 | 95 |
| Mild blackening | 190 | 3.45 ± 2.88 | 12.38 ± 14.2 | 13.38 ± 15.8 | 42 | 87 |
| Split | 126 | - | 14.51 ± 13.48 | 13.4 ± 18.72 | 40 | 45 |
| Extreme blackening | 99 | 3.71 ± 2.77 | 10.15 ± 13.93 | 10.97 ± 15.73 | 28 | 42 |
| Merge | 71 | - | 14.93 ± 13.27 | 15.83 ± 16.1 | 19 | 33 |
| Flash expansion | 64 | - | 13.55 ± 15.44 | 1.2 ± 3.54 | 19 | 25 |
| Flock dilution | 62 | - | 25.8 ± 19.8 | 15.02 ± 18.05 | 13 | 29 |
| Cordon | 35 | 5.45 ± 4.8 | 11.91 ± 13.12 | 9.12 ± 10.32 | 11 | 15 |
| Vacuole | 5 | - | 4.09 ± 2.88 | 6.86 ± 9.41 | 2 | 2 |
| Total | 1067 | - | 13.64 ± 15.06 | 11.97 ± 16.29 | 290 | 471 |

**Table s1** Behavioural events in 67 hunting sequences. The frequency, duration, the average time (plus standard deviation) events preceded and followed attacks and the number of events belonging to sequences with successful (n=16) and unsuccessful (n=26) predation are shown.

**5 Generalised Linear Models on the probability of escape responses**

| Probability flash expansion within 5 seconds after an attack | | | | | | | |
| --- | --- | --- | --- | --- | --- | --- | --- |
| Model | **df** | **AIC_i_** | **Δ_i_(AIC)** | **L_i_** | **W_i_(AIC)** | **acc w_i_(AIC)** | **ER** |
| Attack location + Attack speed | 5 | 210.7 | 0 | 1 | 0.78 | 0.78 | 1 |
| Attack location | 3 | 214.9 | 4.2 | 0.12 | 0.09 | 0.87 | 8.17 |
| Attack speed | 3 | 215.1 | 4.4 | 0.11 | 0.09 | 0.96 | 9.03 |
| Attack location * Attack speed | 9 | 216.5 | 5.8 | 0.06 | 0.04 | 1 | 18.17 |
| 1 (null model) | 1 | 225.7 | 15 | 0 | 0 | 1 | 1808.04 |
| Roosting site | 2 | 227.2 | 16.5 | 0 | 0 | 1 | 3827.63 |

**Table s2** Probability of a flash expansion after an attack: General Linear Models on the probability of a flash expansion occuring 5 seconds after an attack. The AIC scores are shown, as well as the AIC differences Δ_i_(AIC), the likelihood of a model Li, the Akaike weight Wi(AIC), the accumulative Akaike weight acc Wi(AIC) and the Evidence Ratio ER. The explanatory factors tested included attack type, being either repeated attacks or single attacks, attack location, depicting the relative location to the flock, attack speed (depicting the relative speed at which an attack was conducted) and roosting site.

| Probability wave events within 5 seconds before and after an attack | | | | | | | |
| --- | --- | --- | --- | --- | --- | --- | --- |
| Model | **df** | **AIC_i_** | **Δ_i_(AIC)** | **L_i_** | **W_i_(AIC)** | **acc w_i_(AIC)** | **ER** |
| Attack speed | 3 | 229.4 | 0 | 1 | 0.43 | 0.43 | 1 |
| Attack location + Attack speed | 5 | 229.6 | 0.2 | 0.9 | 0.39 | 0.83 | 1.11 |
| Attack location * Attack speed | 9 | 232.5 | 3.1 | 0.21 | 0.09 | 0.92 | 4.71 |
| Attack location | 3 | 233.9 | 4.5 | 0.11 | 0.05 | 0.96 | 9.49 |
| Attack type | 3 | 236.2 | 6.8 | 0.03 | 0.01 | 0.98 | 29.96 |
| 1 (null model) | 2 | 236.7 | 7.3 | 0.03 | 0.01 | 0.99 | 38.47 |

**Table s3** Probability of a wave event before and after an attack: General Linear Models on the probability of a flash expansion occuring 5 seconds after an attack. The AIC scores are shown, as well as the AIC differences Δ_i_(AIC), the likelihood of a model Li, the Akaike weight Wi(AIC), the accumulative Akaike weight acc Wi(AIC) and the Evidence Ratio ER. The explanatory factors tested included attack type, being either repeated attacks or single attacks, attack location, depicting the relative location to the flock, attack speed (depicting the relative speed at which an attack was conducted) and roosting site.

| Probability blackening within 5 seconds before and after an attack | | | | | | | |
| --- | --- | --- | --- | --- | --- | --- | --- |
| Model | **df** | **AIC_i_** | **Δ_i_(AIC)** | **L_i_** | **W_i_(AIC)** | **acc w_i_(AIC)** | **ER** |
| 1 (null model) | 1 | 205.6415 | 0 | 1 | 0.29 | 0.29 | 1 |
| Attack type | 2 | 205.6517 | 0.01 | 0.99 | 0.29 | 0.41 | 1.01 |
| Attack speed | 3 | 207.167 | 1.53 | 0.47 | 0.13 | 0.32 | 2.14 |
| Roosting site | 2 | 207.2605 | 1.62 | 0.45 | 0.13 | 0.46 | 2.25 |
| Attack location | 3 | 208.297 | 2.66 | 0.27 | 0.08 | 0.53 | 3.77 |
| Attack location + Attack speed | 5 | 208.413 | 2.77 | 0.25 | 0.07 | 1 | 4 |

**Table s4** Probability of blackening before and after an attack: General Linear Models on the probability of a flash expansion occuring 5 seconds after an attack. The explanatory factors included attack type, speed and location and the roosting site. The AIC scores are shown, as well as the AIC differences Δ_i_(AIC), the likelihood of a model Li, the Akaike weight Wi(AIC), the accumulative Akaike weight acc Wi(AIC) and the Evidence Ratio ER.

The explanatory factors tested included attack type, being either repeated attacks or single attacks, attack location, depicting the relative location to the flock, attack speed (depicting the relative speed at which an attack was conducted) and roosting site.

| Probability split within 5 seconds before and after an attack | | | | | | | |
| --- | --- | --- | --- | --- | --- | --- | --- |
| Model | **df** | **AIC_i_** | **Δ_i_(AIC)** | **L_i_** | **W_i_(AIC)** | **acc w_i_(AIC)** | **ER** |
| Roosting site | 2 | 223.2 | 0 | 1 | 0.73 | 0.73 | 1 |
| 1 (null model) | 1 | 226.9 | 3.7 | 0.16 | 0.11 | 0.84 | 6.36 |
| Attack type | 2 | 227.9 | 4.7 | 0.1 | 0.07 | 0.91 | 10.49 |
| Flock size | 3 | 230.2 | 7 | 0.03 | 0.02 | 0.93 | 33.12 |
| attack speed | 3 | 230.7 | 7.5 | 0.02 | 0.02 | 0.95 | 42.52 |
| attack location + speed | 5 | 230.7 | 7.5 | 0.02 | 0.02 | 0.97 | 42.52 |
| attack location * speed | 9 | 230.7 | 7.5 | 0.02 | 0.02 | 0.98 | 42.52 |
| attack location | 3 | 230.7 | 7.5 | 0.02 | 0.02 | 1 | 42.52 |

**Table s5** Probability of splitting before and after an attack: General Linear Models on the probability of a flash expansion occuring 5 seconds after an attack. The AIC scores are shown, as well as the AIC differences Δ_i_(AIC), the likelihood of a model Li, the Akaike weight Wi(AIC), the accumulative Akaike weight acc Wi(AIC) and the Evidence Ratio ER. The explanatory factors tested included attack type, being either repeated attacks or single attacks, attack location, depicting the relative location to the flock, attack speed (depicting the relative speed at which an attack was conducted) and roosting site.

**6 Online resources**

**Online Resource 1:** Video footage of the hunting sequence at Eur that was used for the time series analysis shown in figure s1.

**Online Resource 2:** Video footage of a flock of starlings under attack by a peregrine falcon. This footage depicts the falcon employing repeated attacks on a flock, with the flock showing resilience against being split. Attacks occur at 7, 10, 14, 16, 18 and 20 seconds.

**References**

Abràmoff MD, Magalhães PJ, Ram SJ (2004) Image processing with imagej. Biophotonics Int 11:36–42

Schneider CA, Rasband WS, Kevin W, Eliceiri KW (2012) Nih image to imagej: 25 years of image analysis. Nat methods, 9:671–675
